# A 3D In Vivo Model for Studying Human Renal Cystic Tissue and Mouse Kidney Slices

**DOI:** 10.3390/cells11152269

**Published:** 2022-07-22

**Authors:** Eva-Marie Bichlmayer, Lina Mahl, Leo Hesse, Eric Pion, Victoria Haller, Andreas Moehwald, Christina Hackl, Jens M. Werner, Hans J. Schlitt, Siegfried Schwarz, Philipp Kainz, Christoph Brochhausen, Christian Groeger, Felix Steger, Oliver Kölbl, Christoph Daniel, Kerstin Amann, Andre Kraus, Björn Buchholz, Thiha Aung, Silke Haerteis

**Affiliations:** 1 Institute for Molecular and Cellular Anatomy, University of Regensburg, 93053 Regensburg, Germany; eva-marie.bichlmayer@stud.uni-regensburg.de (E.-M.B.); lina.mahl@stud.uni-regensburg.de (L.M.); leo-max.hesse@stud.uni-regensburg.de (L.H.); ericjosh.pion@yahoo.com (E.P.); victoria.haller@stud.uni-regensburg.de (V.H.); andreas.moehwald@stud.uni-regensburg.de (A.M.); thiha.aung@th-deg.de (T.A.); 2 Department of Surgery, University Hospital Regensburg, Franz-Josef-Strauss-Allee 11, 93053 Regensburg, Germany; christina.hackl@klinik.uni-regensburg.de (C.H.); jens.werner@ukr.de (J.M.W.); hans.schlitt@klinik.uni-regensburg.de (H.J.S.); 3 KML Vision GmbH, A-8020 Graz, Austria; siegfried.schwarz@kmlvision.com (S.S.); philipp.kainz@kmlvision.com (P.K.); 4 Institute of Pathology, University of Regensburg, 93053 Regensburg, Germany; christoph.brochhausen@ur.de; 5 Department for Radiotherapy, University Hospital Regensburg, Franz-Josef-Strauss-Allee 11, 93053 Regensburg, Germany; christian.groeger@klinik.uni-regensburg.de (C.G.); felix.steger@klinik.uni-regensburg.de (F.S.); oliver.koelbl@ukr.de (O.K.); 6 Department of Nephropathology, Friedrich-Alexander University Erlangen-Nürnberg, 91054 Erlangen, Germany; christoph.daniel@uk-erlangen.de (C.D.); kerstin.amann@uk-erlangen.de (K.A.); 7 Department of Nephrology and Hypertension, University Hospital Erlangen, University of Erlangen-Nuremberg, 91054 Erlangen, Germany; andre.kraus@uk-erlangen.de (A.K.); bjoern.buchholz@uk-erlangen.de (B.B.); 8 Faculty of Applied Healthcare Science, Deggendorf Institute of Technology, 94469 Deggendorf, Germany

**Keywords:** human renal cystic tissue, ADPKD, chorioallantoic membrane (CAM) model, mouse kidney slices, 3D in vivo model, polycystic kidney disease

## Abstract

(1) Background: Autosomal dominant polycystic kidney disease (ADPKD) is a frequent monogenic disorder that leads to progressive renal cyst growth and renal failure. Strategies to inhibit cyst growth in non-human cyst models have often failed in clinical trials. There is a significant need for models that enable studies of human cyst growth and drug trials. (2) Methods: Renal tissue from ADPKD patients who received a nephrectomy as well as adult mouse kidney slices were cultured on a chorioallantoic membrane (CAM) for one week. The cyst volume was monitored by microscopic and CT-based applications. The weight and angiogenesis were quantified. Morphometric and histological analyses were performed after the removal of the tissues from the CAM. (3) Results: The mouse and human renal tissue mostly remained vital for about one week on the CAM. The growth of cystic tissue was evaluated using microscopic and CT-based volume measurements, which correlated with weight and an increase in angiogenesis, and was accompanied by cyst cell proliferation. (4) Conclusions: The CAM model might bridge the gap between animal studies and clinical trials of human cyst growth, and provide a drug-testing platform for the inhibition of cyst enlargement. Real-time analyses of mouse kidney tissue may provide insights into renal physiology and reduce the need for animal experiments.

## 1. Introduction

Out of the group of monogenic disorders resulting in renal cyst development [1], autosomal dominant polycystic kidney disease (ADPKD) represents the most common form of polycystic kidney disease (PKD) [2], with an incidence of 1:400–1:1000 [1]. The disease is characterized by bilateral cysts that usually originate from distal regions of the nephron and the collecting duct, from where they progressively enlarge and spread over time [1,2]. Consequently, ADPKD often leads to chronic kidney disease (CKD) and end-stage renal disease (ESRD) with the necessity for dialysis or kidney transplantation [2,3]. Because ADPKD is a rather slowly progressing disease that often develops over decades, there is a large time span for the potential inhibition or reversal of cyst growth and the subsequent preservation of renal function.

ADPKD is mainly caused by heterozygous mutations of the *PKD1* or *PKD2* genes, which encode for the polycystin-1 (PC1) and 2 (PC2) proteins, respectively [4]. PC1 and PC2 are suspected to form a functional complex located in the primary cilium [5]. There are multiple pathways and signaling molecules that cause phenotypic changes in renal epithelial cells, which ultimately lead to cyst formation. How malfunctioning PKD proteins affect these pathways is not yet fully understood [6]. For the implementation of novel treatment targets and strategies to preserve renal function in patients suffering from ADPKD, it is crucial to identify the cellular mechanisms and understand the pathophysiological cascade involved in the formation and growth of renal cysts in ADPKD.

Cyst progression is accompanied by regional hypoxia, presumably caused by the compression of the peritubular vasculature, which leads to the induction of hypoxia-inducible transcription factor (HIF) 1α. HIF-1α appears to be an important mediator and accelerator of cyst growth, and has a strong cyst-growth-promoting effect in ADPKD mouse models, especially in severe phenotypes of ADPKD. Subsequently, HIF-1α-dependent signaling pathways may qualify as possible therapeutic targets for the treatment of ADPKD [7,8].

Furthermore, there is a broad consensus regarding the important roles that fluid secretion and cell proliferation play in the multidimensional cyst development and enlargement process [6,9]. Enhanced fluid secretion is supposed to be mediated by the cystic fibrosis transmembrane conductance regulator (CFTR), which facilitates cAMP-stimulated chloride secretion in ADPKD cysts [10,11]. The involvement of CFTR in the progression of ADPKD is supported by the increased intracellular cAMP levels in ADPKD cysts that have been identified as a common pathogenic mechanism of the disease [6]. The only medication that has been approved for the treatment of ADPKD so far is tolvaptan^®^, a vasopressin V_2_ receptor antagonist which targets the antidiuretic hormone arginine vasopressin (AVP)-stimulated cAMP production. Multiple studies have demonstrated the effectiveness of tolvaptan^®^ when it comes to the suppression of cAMP production as well as renal cell proliferation [4,12]. Nevertheless, it should be noted that the application of tolvaptan^®^ bears the risk of hepatotoxicity and significant aquaretic adverse events [4].

Although the pathogenic role of cAMP is evident in ADPKD, the role of CFTR is still questionable. Recently, Talbi et al. showed that cAMP-activated fluid secretion through CFTR only contributed to the ADPKD pathophysiology marginally, and the deletion of CFTR was not effective in resolving the cystic phenotype in an adult ADPKD mouse model [11]. However, the exact role of CFTR in human ADPKD remains unclear so far.

Moreover, TMEM16A, a Ca^2+^-activated chloride channel, was identified as a promising pharmacological target [13]. In recent studies, TMEM16A was a significant contributor to cyst growth through epithelial fluid secretion [9,11]. In vivo as well as in vitro models have clearly demonstrated that up-regulated TMEM16A plays an essential role for the progression of ADPKD [9]. Furthermore, the inhibition of TMEM16A through the FDA-approved drugs niclosamide and benzbromarone, as well as the newly discovered highly specific inhibitor Ani9, potently suppressed cyst growth in an orthologous ADPKD mouse model [9].

Despite these promising data, the translation of these findings into recent clinical applications appears to be difficult. Even though there have been multiple preclinical studies that have shown effective targeting of the signaling pathways and molecules in animal models of PKD, the translation of these findings into clinical applications has often failed [14,15].

The chorioallantoic membrane (CAM) model could bridge the gap between preclinical animal studies and clinical trials. The CAM is formed as an extraembryonic membrane within fertilized chicken eggs. It develops as a mesodermal fusion of the allantois and chorion between the third and tenth days of embryonic development [16,17] and mainly functions as the respiratory organ of the chicken embryo [17]. As such, it is connected to the embryonic circulation and comprises a high density of blood vessels that form a dense capillary network [18].

Due to its high level of vascularization, the CAM has been widely utilized as a platform for in vivo studies of vascular development and angiogenesis. It has also gained a lot of attention as a tumor model for the investigation of tumor growth and metastasis, drug distribution and screening, and many further applications since its first implementation by Rous and Murphy in 1911 [17,18,19]. The use of the CAM model has increased because it does not need approval from animal research ethics committees. This has become even more important since the introduction of the 3R (reduction, refinement, replacement) principle, which aims to reform the use of animal models in research [16]. Furthermore, the CAM is characterized by its accessibility and growth-promoting features [17], as well as its lack of a fully developed immune system, which enables the analysis of different tumor tissues due to low rejection rates [16].

Taking these facts together, we hypothesized that the CAM could provide a vascular matrix that would enable the growth and assessment of human cystic kidney tissue for the study of cyst growth as well as mature mouse kidneys for the investigation of complex renal processes in vivo. Whole metanephric mouse kidneys can be cultured ex vivo on filters for several days. Due to their small size and texture, the vitality can be maintained simply by diffusion with an adapted cell culture medium [20]. However, adequate models for the assessment of mature mouse kidneys have not yet been described to our knowledge. Adult mouse kidneys cannot be supplied with essential oxygen and nutrients via diffusion, instead requiring a more complex methodology. Again, the CAM may provide a platform that would enable the maintenance and analysis of mature kidneys, which would otherwise only be possible by elaborate means such as intravital microscopy.

We have long-standing expertise using the 3D in vivo CAM model for human tumor research, and we have established the CAM model for the investigation of the effects of different therapies (chemotherapy, radiation therapy) on tumor growth and angiogenesis [21,22,23,24,25,26].

The aim of the present study was to establish a platform for the successful translation from preclinical animal models to clinical studies regarding the treatment of ADPKD. Therefore, we put forward a well-rounded protocol for the analysis of ADPKD patient tissue. In addition, this 3D in vivo model enables the cultivation and analysis of adult mouse kidney slices.

## 2. Materials and Methods

### 2.1. Chorioallantoic Membrane (CAM) Model

**Figure 1:**
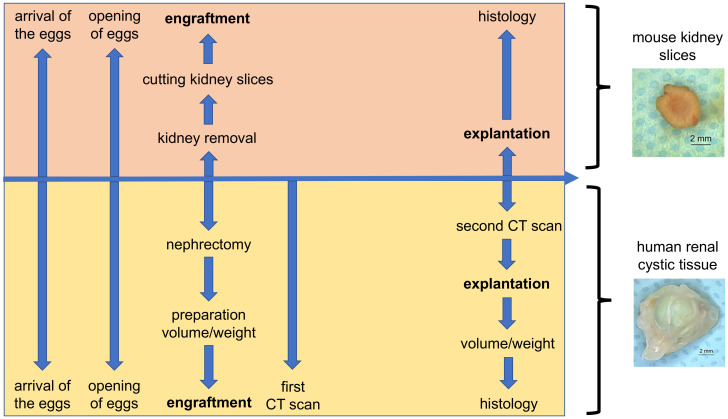
Schematic depiction of the chorioallantoic membrane (CAM) model protocol for the investigation of human renal cystic tissue and mouse kidney slices.

The CAM model was performed as previously described [24,25,26,27]. Upon arrival, chicken eggs were disinfected with ethanol (70%) and placed in a ProCon egg incubator (Grumbach, Asslar, Germany) at 37.8 °C and 63% humidity in combination with hourly rotation. After four days of incubation, a window of approximately 1 cm^2^ was cut into the shell of the fertilized eggs and sealed with Leukosilk^®^. The eggs were engrafted with a kidney slice from a mouse kidney or with human renal cystic tissue between the 7th and 10th days of embryonic development. For the engraftment process, the tissue was gently moved on the CAM with pincers after the vessels were roughened to ensure that the tissues were fixed on the CAM. During the period of investigation, the engrafted eggs were checked for vitality once per day. After removing the tissue between the 15th and 16th days of incubation, the eggs were immediately placed in liquid nitrogen. Tissues were embedded in paraformaldehyde (PFA) and subjected to further histological examinations (Figure 1).

### 2.2. Cultivation of Mouse Kidney Slices on the CAM

After removing the kidneys from adult mice (C57BL6J), the kidneys were put in petri dishes with cooled phosphate-buffered saline (PBS). All animal experiments were approved by local government authorities and were performed in accordance with the US National Institutes of Health Guide for the Care and Use of Laboratory Animals. The kidneys were cut into thin slices, either mechanically with the help of a vibratome (600 µm) or manually using a razor blade (about 600 µm). Directly after engraftment, the kidney slices were covered with an autoclaved fleece for immobilization. For an abundant supply of nutrients, the mouse kidney slices were treated with daily applications of 100 µL of RPMI-1640 medium (Sigma-Aldrich, Taufkirchen, Germany) until they were removed from the CAM after five to eight days of examination.

### 2.3. Cultivation of Tissue from Human Cystic Kidneys on the CAM

Human renal cystic tissue was obtained from ADPKD patients who received a nephrectomy at the University Hospital Regensburg. Overall, the renal cystic tissue of eight different nephrectomies was investigated. Written consent was obtained from all patients and experiments were approved by the ethics committee of the University of Regensburg (no. 20-1886-101).

#### 2.3.1. Preparation of Cystic Tissue

**Figure 2:**
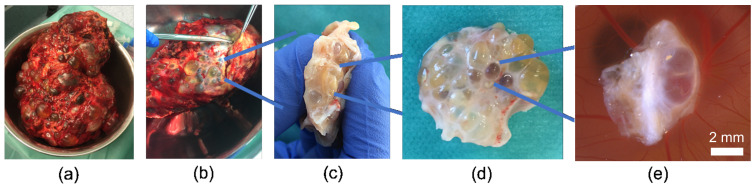
Workflow from the nephrectomy to the engraftment on the 3D in vivo model; (**a**) entire human kidney from a patient suffering from ADPKD; (**b**) dissection of capsula adiposa; (**c**,**d**) dissected cystic tissue; and (**e**) renal cystic tissue implanted on the CAM.

After the removal of a polycystic kidney from a patient, small renal cystic tissue pieces containing predominantly microcysts were dissected and stored in tubes filled with culture medium until further processing was carried out (Figure 2a–d). After washing the cystic tissue in PBS, small pieces of cystic tissue were prepared and grafted onto the CAM (Figure 2e). Microscopic photographs of the CAM and the engrafted renal cysts were taken every second day over the course of one week with a Leica M205A microscope (Leica Microsystems, GmbH, Wetzlar, Germany) and a Leica MC170 HD camera. The cystic tissue was treated with daily applications of 100 µL of 0.9% sodium chloride or distilled water, particularly to prevent the parts of the tissue that were exposed to air and lacked a direct connection to the CAM from drying out.

#### 2.3.2. Ex Ovo Volume and Weight Measurement

To monitor volume and weight, the human renal cysts were measured ex ovo before engraftment and after removal from the CAM. The 3D microscope Keyence VHX-7000 (Keyence Deutschland GmbH, Neu-Isenburg, Germany), which utilizes the so-called “depth from defocus” technology, was used to perform volume measurements [23]. A standard laboratory balance was used for the evaluation of the weight of the cystic tissue.

#### 2.3.3. In Ovo Computed Tomographic (CT)-Based Volume Measurements

For in ovo volume measurements of the human renal cystic tissue, computed tomographic (CT) scans were performed for 36 eggs, one or two days after engraftment and hours before the removal of the cystic tissue. CT scans were taken using the Canon Aquilion LB TSX-201A patient CT machine (Canon Medical Systems GmbH, Neuss, Germany) at the Department for Radiotherapy of the University Hospital Regensburg. Images in the transverse, sagittal, and coronal planes of the eggs were obtained using a soft-tissue-based scan program specifically adapted for the analysis of small tissue structures in the CAM model. Subsequently, the reconstructed CT datasets were uploaded into the radiotherapeutic planning software Monaco 5.51 (Elekta, Stockholm, Sweden), where cystic tissue was manually contoured in each layer of the datasets using different window and level sets (e.g., adapted soft tissue or lung window). Based on these contoured structures, the software calculated the volume of the cystic tissue.

#### 2.3.4. Measurement of Angiogenesis with LSCI and the CAM Assay Application

For the assessment of the process of angiogenesis on the CAM and the perfusion of the cystic tissue, we utilized laser speckle contrast imaging (LSCI) at the beginning and after renal cystic tissue growth. LSCI is a semi-quantitative method which can be used to measure microcirculatory blood perfusion based on the fluctuations of the contrast in a speckle pattern that is caused by the backscattering of laser arrays due to the movement of erythrocytes in the blood vessels. The PeriCAM perfusion speckle imager (PSI) system high resolution (HR) model (PERIMED, Netherlands) was used for LSCI measurements adjacent to the previously described protocol, and the average perfusion rate was calculated by the PimSoft version 1.5 software [25]. Minor deviations from the previously described protocol included an increased sampling frequency of 44 Hz, which increased the number of images that were used to calculate an average for one measurement to 88. This resulted in an effective frame rate of 0.5 images/s. Furthermore, the number of single consecutive measurements for which the embryo needed to remain static was increased to ten, which equaled 20 s.

The perfusion images taken during the LSCI measurements were evaluated after all measurements were conducted, and the minimum cut-off for all measurements was set to 700 perfusion units (PU), which resulted in a threshold of 700–3000 PU. To ensure an accurate depiction of the perfusion, the results were corrected by subtracting the area of the CAM that was covered by the cystic tissue, and hence could not be measured by LSCI, from the overall image in accordance with our previous description [25]. To assure comparability of the two measurements, which differed in the total measured area (~110 mm^2^ for the first and ~90 mm^2^ for the second measurement) of each egg, the measured area of the second measurement, which resulted after the application of the cut-off, was multiplied by the division of the total individual area of the first measurement by the total individual area of the second measurement (area × (~110 mm^2^/~90 mm^2^).

**Figure 3:**
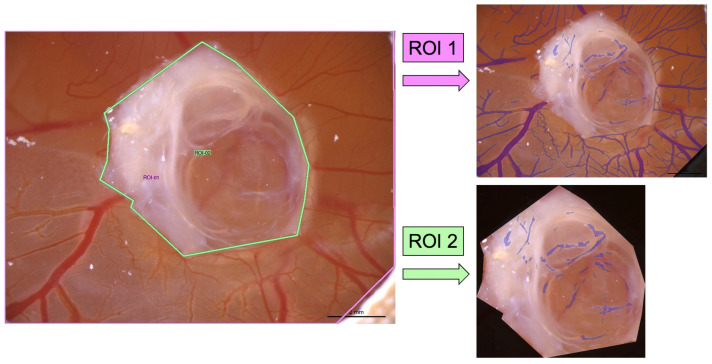
Definition of regions of interest (ROIs) in the CAM assay application. The first ROI (ROI 1) was defined as the total CAM with the engrafted cystic tissue samples. The second ROI (ROI 2) only contained the cystic tissue. The values of ROI 2 were subtracted from the results of ROI 1 to ensure that false positive vessel detections in the cystic tissue were excluded from the analysis.

In addition to the LSCI measurements, the blood vessels of the CAM were also analyzed using the CAM Assay application of the IKOSA platform (KML Vision, Graz, Austria), which is an image analysis algorithm that was specifically developed for the ex ovo CAM model and enables the quantification of blood vessels (total vessel length, total vessel area, number of branching points, and mean vessel thickness) in microscopic images based on artificial intelligence. The software calculated these parameters within seconds for the quantification of the angiogenesis of the exposed CAM. The CAM assay application v3.0.0 was used to assess the blood vessels on the day of the engraftment of human cystic tissue and before the removal of the samples from the CAM after a week. Only images with identical exposure times were used to compare the angiogenesis measurements. False positive vessel detections by the application in the engrafted cystic tissue were excluded from the analysis using the region of interest (ROI) feature of the software. Therefore, two ROIs were defined and the values of the second ROI, which only contained the cystic tissue, were subtracted from the overall results of the ROI that included the CAM and the engrafted ADPKD tissue sample (Figure 3).

### 2.4. Immunohistochemistry and Antibodies

#### 2.4.1. Paraffin Sections

After the mouse and human ADPKD tissue samples were removed from the CAM, they were fixed in 4% PFA in PBS with a pH of 7.4. After washing them three times in PBS, the specimens were dehydrated in a 26-h procedure using an automatic tissue infiltration machine and were then embedded in paraffin. The paraffin blocks were finally cut into 6 μm-thin parts with a microtome.

#### 2.4.2. Staining and Immunohistochemistry

Hematoxylin and eosin staining (H&E staining) was performed as a routine staining procedure for all specimens. Prior to staining, the slides were first deparaffinized in xylene, hydrated in a graded alcohol series, and afterwards stained with H&E according to the standard protocol.

For the detection of HIF-1α, polyclonal rabbit anti-HIF-1α (Cayman Chemicals, Ann Arbor, MI, USA; 1:5000) primary antibodies were used. A catalyzed signal amplification system and a biotinylated secondary anti-rabbit IgG (Vector, Burlingame, CA, USA; 1:500) were used according to the manufacturer’s instructions. Sections were counterstained with hematoxylin. Immunofluorescence was performed using the following primary antibodies: Primary cilia were stained with monoclonal mouse anti-acetylated tubulin antibodies (Sigma; 1:200); E-cadherin was stained with monoclonal rabbit anti-E-cadherin antibodies (Cell Signaling, Danvers, MA, USA; 1:200); phosphorylated (Ser235/236) S6 kinase beta-1 was detected using polyclonal rabbit anti-pS6k1 antibodies (Cell Signaling; 1:200); Dolichos biflorus agglutinin (DBA) binding was visualized using fluorescein-labeled DBA (Vector; 1:500); chicken and mouse CD31 was detected using a monoclonal rat anti-mouse CD31 antibody (Dianova GmbH, Hamburg, Germany; 1:50); human CD31 was detected using a monoclonal mouse anti-human CD31 antibody (DAKO GmbH, Glostrup, Denmark; 1:100); human macrophages were detected using a monoclonal anti-CD68 antibody (DAKO; 1:100); proliferating cells were detected using a monoclonal mouse antibody directed against proliferating cell nuclear antigen (PCNA) (DAKO; 1:200); megalin was detected using a monoclonal mouse anti-megalin antibody (OriGene Technologies GmbH, Herford, Germany; 1:100); the intermediary filament vimentin was detected using a monoclonal mouse anti-human vimentin antibody (DAKO, 1:200); uromodulin was detected using a mouse monoclonal antibody directed against uromodulin (Santa Cruz Biotechnology, Santa Cruz, CA, USA; 1:200); apoptotic cells were detected using a polyclonal rabbit antibody directed against cleaved caspase 3 (Cell Signaling Technology Europe, Frankfurt am Main, Germany; 1:200); and the sodium/calcium exchanger was detected in mouse kidneys as a marker of distal tubules using a mouse monoclonal antibody directed against the Na/Ca exchanger (Swant, Burgdorf, Switzerland; 1:200). After washing with 50 mM Tris and 150 mM NaCl supplemented with 0.05% Tween 20 (TBST), bound primary antibodies were detected with the appropriate fluorescence-labeled secondary antibodies: Alexa Fluor 555-labeled donkey anti-mouse IgG antibody (Invitrogen, Waltham, MA, USA; 1:1000); Alexa Fluor 555-labeled donkey anti-rabbit IgG antibody (Invitrogen; 1:1000); Alexa Fluor 488-labeled goat anti-mouse IgG antibody (Invitrogen; 1:500); Alexa Fluor 488-labeled donkey anti-rat IgG antibody (Invitrogen, 1:500); Alexa 568-labeled donkey anti-rabbit IgG antibody (Invitrogen, 1:500); Alexa 647-labeled donkey anti-rabbit IgG antibody (Invitrogen, 1:500); Alexa 568-labeled donkey anti-mouse IgG antibody (Invitrogen, 1:500); Alexa 488-labeled goat anti-mouse IgG3 antibody (Dianova GmbH, Hamburg, Germany; 1:500); Alexa 647-labeled donkey anti-mouse IgG antibody (Invitrogen, 1:500); Alexa 647-labeled donkey anti-mouse IgG1 antibody (Invitrogen, 1:500); Alexa 568-labeled goat anti-rabbit IgG2a antibody (Invitrogen, 1:500); and Alexa 555-labeled donkey anti-rabbit IgG antibody (Invitrogen, 1:500). After the removal of non-bound secondary antibodies by washing with TBST, the tissue sections were covered with DAPI containing mowiol.

Signals were either analyzed with a DM6000B fluorescence microscope (Leica, Wetzlar, Germany), with photographs taken using a Leica DFC 450C camera, or using a confocal laser scanning microscope LSM710 together with the Zen2009 software (Zeiss, Oberkochen, Germany).

### 2.5. Statistical Analysis

The creation of graphs and the analyses were carried out using the GraphPad Prism 8 software.

## 3. Results

### 3.1. Cultivation of Adult Mouse Kidney Slices on the CAM

**Figure 4:**
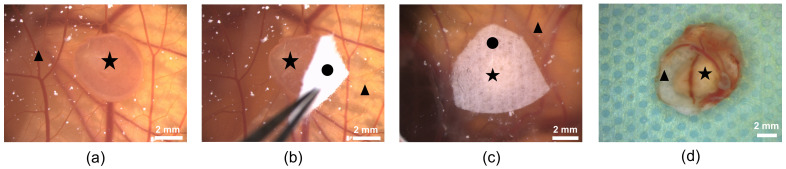
Mouse renal tissue on the CAM; (**a**) kidney slice after engraftment; (**b**) covering of the tissue with a piece of fleece; (**c**) kidney slice covered with fleece; and (**d**) kidney slice with surrounding CAM and distinct blood vessels after removal. (▲: CAM with blood vessels; ★: kidney slice; ●: fleece).

Until now, it has not been possible to culture sections from adult mouse kidneys using in vivo models. Therefore, we successfully cultured and analyzed 41 mouse kidney slices from seven different mice on the CAM (Figure 4). The adult mouse kidney slices appeared to remain vital on the CAM for the time of the experiment (up to one week) (Figure 4a–d).

**Figure 5:**
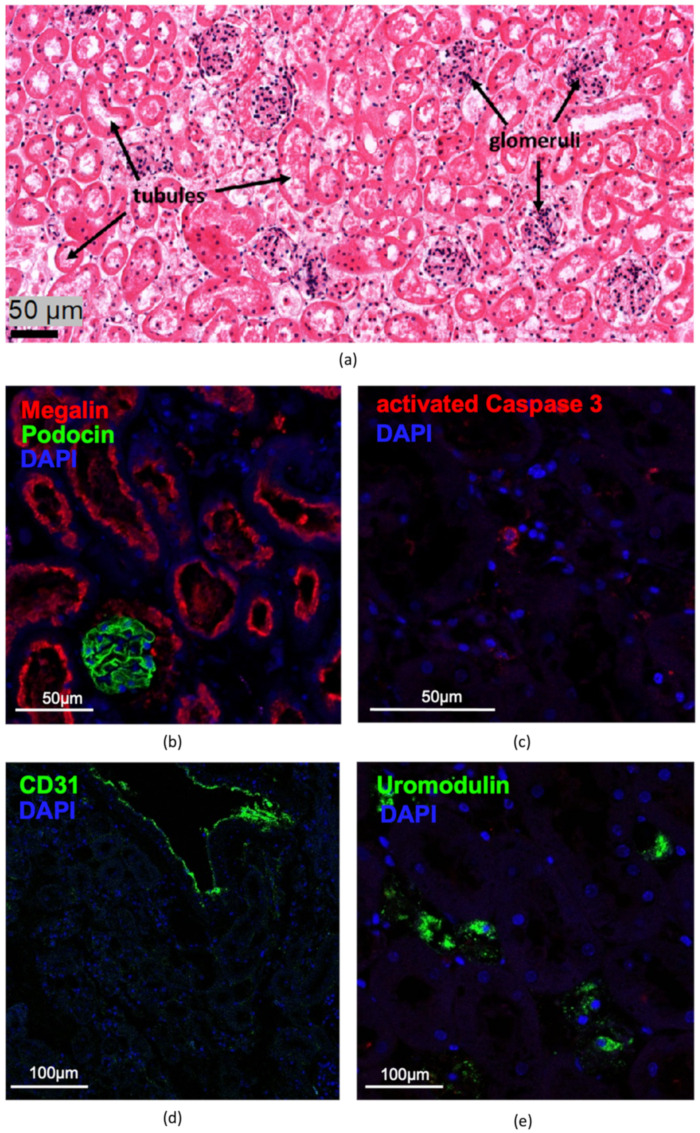

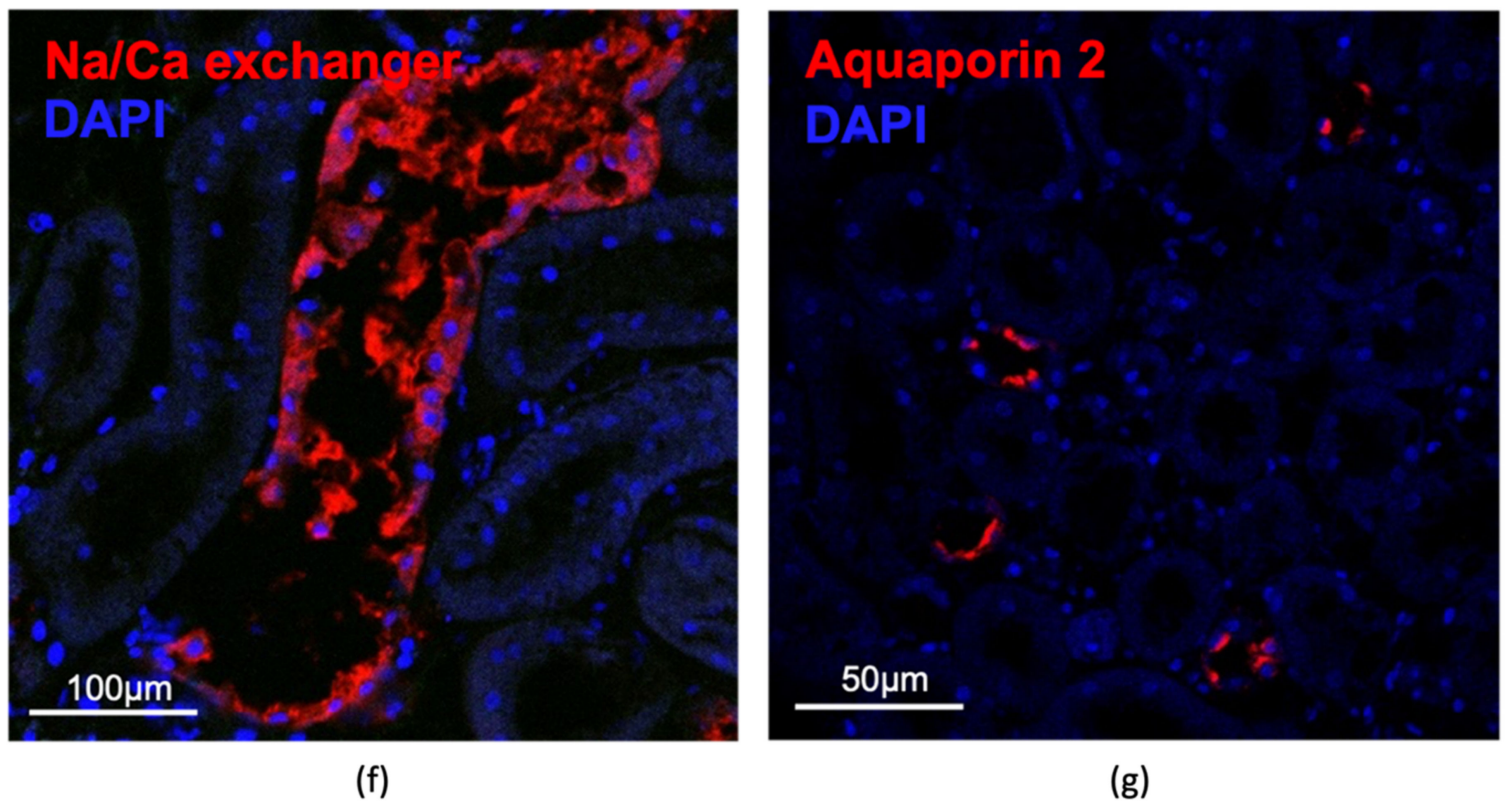
Histological and immunofluorescence analyses of kidney slices after removal from the CAM; (**a**) H&E staining; (**b**) proximal tubules and podocytes stained with megalin (red) and podocin (green), respectively; (**c**) only single apoptotic-activated caspase 3-positive cells could be detected; (**d**) detection of CD31 in renal vessels; (**e**) uromodulin- and (**f**) Na/Ca exchanger-positive cells showed damage, while (**g**) aquaporin 2-positive cells were preserved. Nuclei were stained with DAPI (blue).

The use of a fleece to cover the kidney slices on the CAM enabled the immobilization of the renal tissue and the engraftment of the kidney slices onto the CAM. Furthermore, we suspected that it would be a reservoir for nutrients, which were supplied to the tissue by treatment with an RPMI medium. We observed that the fleece-covered slices did not change their macroscopical appearance (Figure 4) or 3D structure during the investigation time, and that blood vessels localized around the renal tissue increased in number and thickness. This indicated successful engraftment and maintenance of the kidney slices on the membrane over time, and was also confirmed by H&E staining, which seemed to show vital structures that depicted intact tubules and glomeruli (Figure 5a). The subsequent immunofluorescence microscopic analyses confirmed the presence of typical renal markers, such as podocin for podocytes (Figure 5b) and megalin for proximal tubules (Figure 5b), and a low rate of apoptotic cells detected by activated caspase 3 (Figure 5c). However, no proliferating cells were detected when staining for proliferating cell nuclear antigen (PCNA) (data not shown). In addition, CD31-positive endothelial cells were detected in arterioles, but not in peritubular capillaries after tissue removal (Figure 5d). In contrast, the staining of uromodulin (Figure 5e) as a marker of the thick ascending limb and the Na/Ca exchanger (Figure 5f) as a marker of distal tubules showed damage in these two tubule segments. The collecting ducts, assessed by aquaporin 2 staining, were intact (Figure 5g).

### 3.2. Growth of Human Cystic Kidney Tissue on the CAM

**Figure 6:**
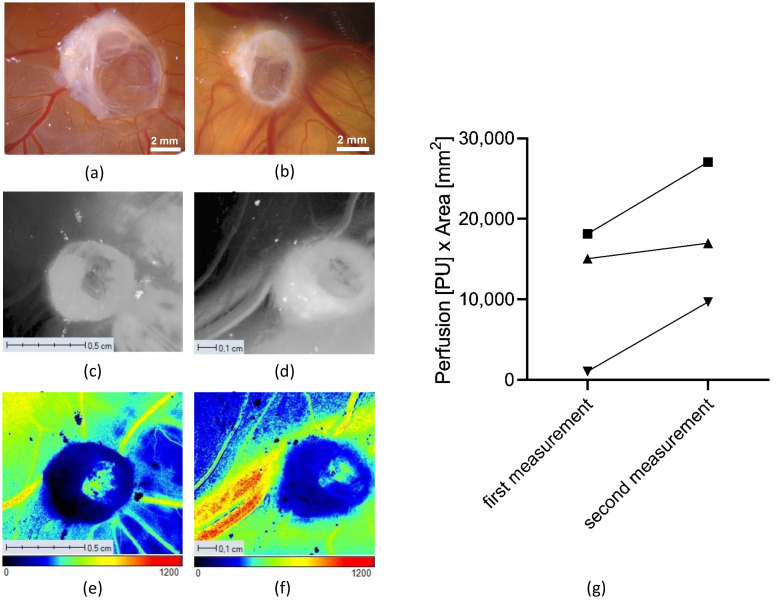
Angiogenesis measurements. Microscopic pictures of the CAM with renal cystic tissue after engraftment (**a**) and before removal (**b**). Monochrome intensity images of the same tissue (**c**,**d**) and LSCI perfusion images (**e**,**f**) (a color bar illustrates the perfusion scale) five days after engraftment (**c**,**e**) and on the day of removal (**d**,**f**). The diagram (**g**) shows an increase in the perfusion of three different cystic tissues between the two measurement days (*n* = 3).

The second part of the study focused on the cultivation of human polycystic kidney tissue on the CAM. To mimic the in vivo situation, we grafted freshly excised renal cystic tissue directly onto the CAM, thereby maintaining the heterogeneity and the whole 3D structure of the renal cystic tissue, which are lost in homogenous cultures of renal cells. A total of 153 eggs with cystic material from eight nephrectomies were analyzed. The tissue was obtained from patients of different ages and genders. Furthermore, different parts of the same tissue were engrafted onto a varying number of eggs, which contributed to the overall heterogeneity of the analyzed tissue. After a cultivation period of approximately one week, the engrafted tissue samples remained vital and maintained their 3D structure for the most part, according to our macroscopical images (see Figure 2, Figure 4, and Figure 6). The images were taken during the growth on the membrane, in addition to further microscopic analyses after tissue removal.

#### 3.2.1. Angiogenesis Measurements

We observed a clear macroscopic increase in the blood perfusion of human cystic samples on the CAM, and subsequently analyzed the angiogenesis of the engrafted human cystic renal tissue samples with LSCI during the cultivation period on the CAM using the PeriCam PSI system HR model.

As indicated by an increase in the diameter of the blood vessels in the photographs taken during the LSCI measurements, the perfusion of the blood vessels of the CAM increased during the one-week cultivation period (Figure 6a–f). The overall perfusion was calculated by multiplying the measured perfusion units (PU) with the measured perfusion area (mm^2^), which resulted in an increase in the microcirculatory perfusion after the growth period on the CAM (Figure 6g). This could be attributed to both an increase in the number of blood vessels and the increased perfusion of the already existing blood vessels that were detected by the LSCI.

**Figure 7:**
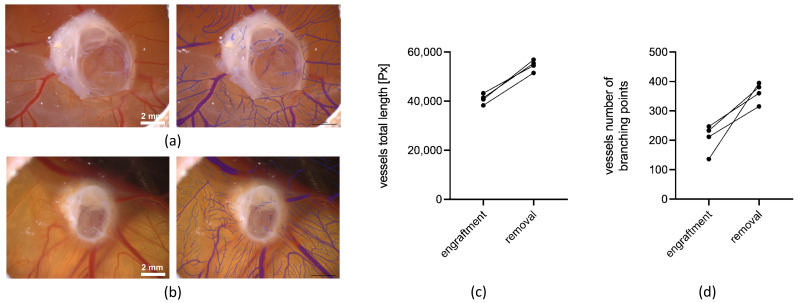
Analysis of the angiogenesis induced by renal cystic tissue on the CAM using the CAM assay application v3.0.0 and microscopic images taken with the Leica M205A microscope after the engraftment of the renal cystic tissue ((**a**), **left**) and prior to the removal ((**b**), **left**). Blood vessels which are marked blue in the microscopic images were recognized by the algorithm ((**a**,**b**), **right**). Analysis of the blood vessels of the CAM (**c**) showed an increase in the total vessel length during the experiments (*n* = 4). Quantification of the number of branching points (**d**) showed an increase in vessel ramification after one week of growth on the CAM (*n* = 4).

In addition to the LSCI measurements, the CAM assay application v3.0.0 was used to analyze the blood vessels of the CAM regarding the preferred angiogenic parameters, such as the total vessel length and the number of branching points of the blood vessels (Figure 7). In contrast to the LSCI measurements, vessels of a smaller size could be much more accurately quantified with this algorithm. The analysis was carried out based on microscopic photographs of the CAM taken with the Leica M205A microscope after the engraftment and before the removal of the cystic tissues (Figure 7a,b left). For our evaluation of angiogenesis with the CAM assay application v3.0.0, we utilized the total vessel length and the number of branching points, as we found these parameters to be the most robust when applying the methodology to the CAM. The total length of the CAM vessels increased during the cultivation period of the cystic tissue (Figure 7c). The number of branching points also showed an increase from the time of engraftment to the time of the removal of the cystic tissue (Figure 7d).

#### 3.2.2. Ex Ovo 3D Volume Measurements and Weight Measurements of Human Renal Cystic Tissue

**Figure 8:**
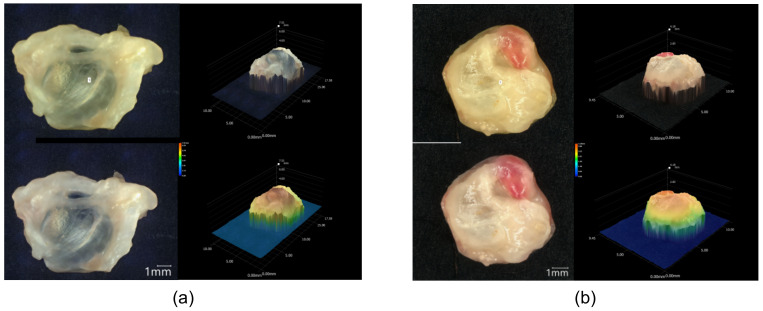

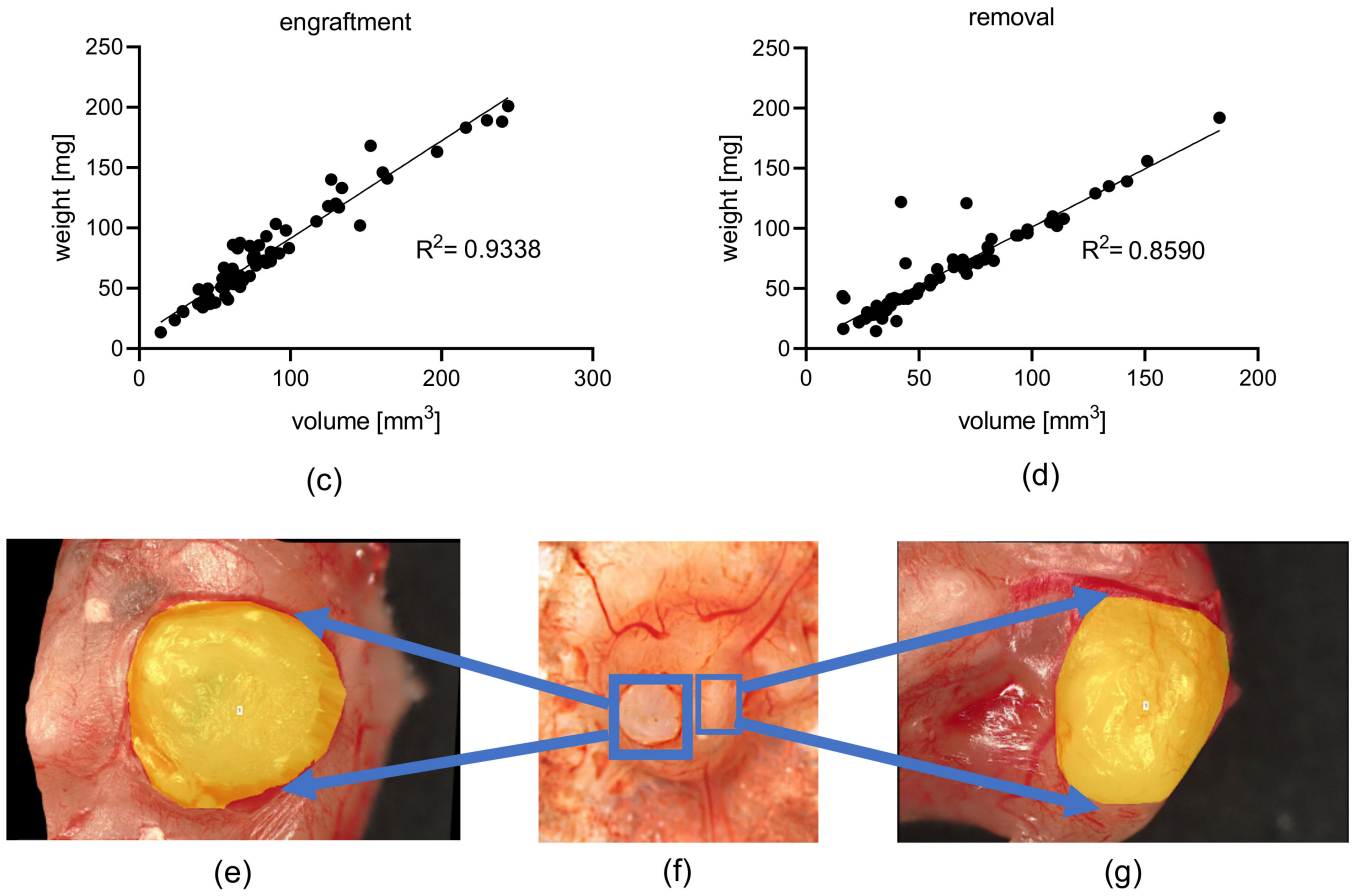
Three-dimensional volume measurements. Measurement of cystic tissue before engraftment (**a**) and after removal from the CAM (**b**) using a Keyence VHX-7000 microscope, including 2D and 3D images of the cystic tissue with volume measurements of the manually defined structure of interest. Linear regression analysis of weight (mg) obtained with a precision balance and volume (mm^3^) obtained with 3D microscopy of the cystic tissues before engraftment and after removal (**c**,**d**). Each point represents a single measurement of weight and volume obtained from three nephrectomies (*n* = 66). The calculated linear regression is depicted by the continuous line. Microscopic image of the bottom of a cystic tissue (**f**) with visible newly formed cysts magnified in (**g**,**e**) and marked with yellow color.

To monitor the volume of the human renal cystic tissue, volume measurements as well as weight measurements of the tissue samples were conducted ex ovo before the engraftment and after the removal of the cystic tissue from the CAM (Figure 8). The volume measurements of ADPKD-patient-derived tissue using the Keyence VHX-7000 microscope proved to be efficient for the assessment of ex ovo volumetric changes based on a correlation between tissue weight and volume (Figure 8a–d).

Moreover, the sequential volume measurements allowed the detection of newly formed cysts within a one-week growth period on the CAM. After removing the tissue, we observed the formation of new cysts on the bottom of the renal tissue (Figure 8e–g).

#### 3.2.3. Comparison of In Ovo (CT) and Ex Ovo (3D) Measured Volumetric Data

**Figure 9:**
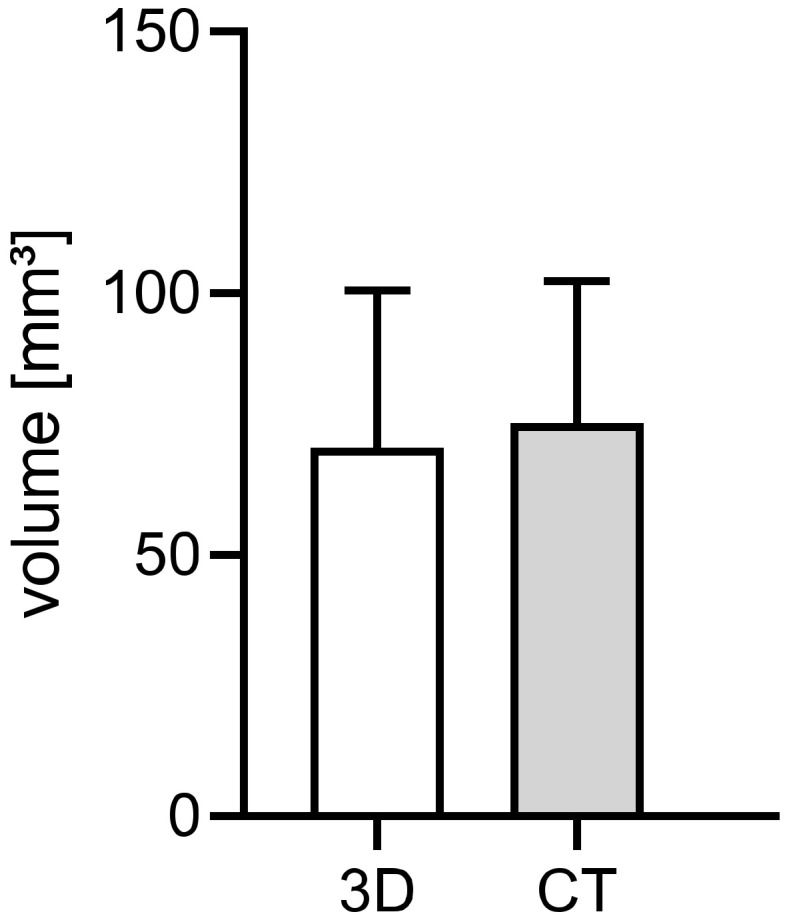
Comparison of ex ovo and in ovo measured volumetric data after engraftment. For a comparison of the CT-based volume measurement method (grey bar) to the three-dimensional microscopic measurement method (white bar), data from three nephrectomies were evaluated (*n* = 36).

For the assessment of the volume of engrafted cystic tissue samples, we also performed CT-based volume measurements of 36 cystic tissues. The CT scans enabled an in ovo visualization and characterization of the cystic tissue based on the high grey-scale value of the tissue. To evaluate the accuracy of the CT-based approach for the volume measurements of human renal cystic tissue in the CAM model, the acquired data were compared to the results of the microscopic ex ovo volume measurements. The comparison of data acquired at the beginning of the cultivation period of the ADPKD tissue on the CAM resulted in a mean deviation of 11% from the 3D-volumetric measurements (Figure 9).

**Figure 10:**
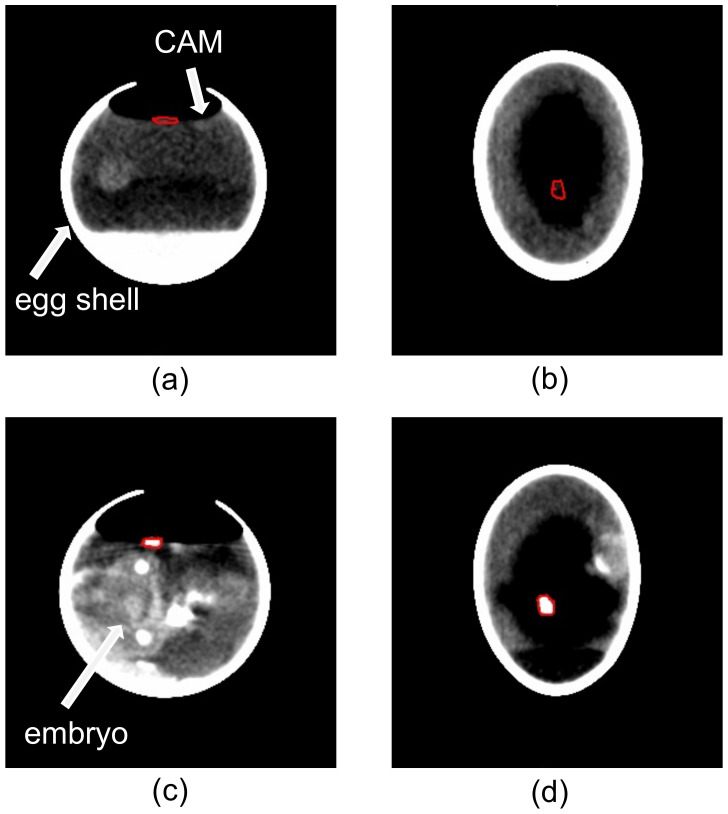
CT-based volume measurement of a cystic tissue depicted in the cyst window (*n* = 36). The cystic tissue is marked by the red border in each image. The chicken egg with engrafted cystic tissue is shown one day after engraftment in the transverse (**a**) and coronal (**b**) planes without any signs of calcifications. A significant increase in hyperdense structures in the cystic tissue was visible in the transverse (**c**) and coronal (**d**) planes of the second measurement, which took place hours before removal of the cystic tissue.

Particularly interesting was the fact that there was a strong increase in calcifications for most of the cystic tissue samples between the first and second CT scan (Figure 10). The calcifications were similar to the density of the developing bone structures of the chicken embryo underneath the CAM.

#### 3.2.4. Characterization of the Human Renal Cystic Tissue after Growth on the CAM (Histological Analysis)

**Figure 11:**
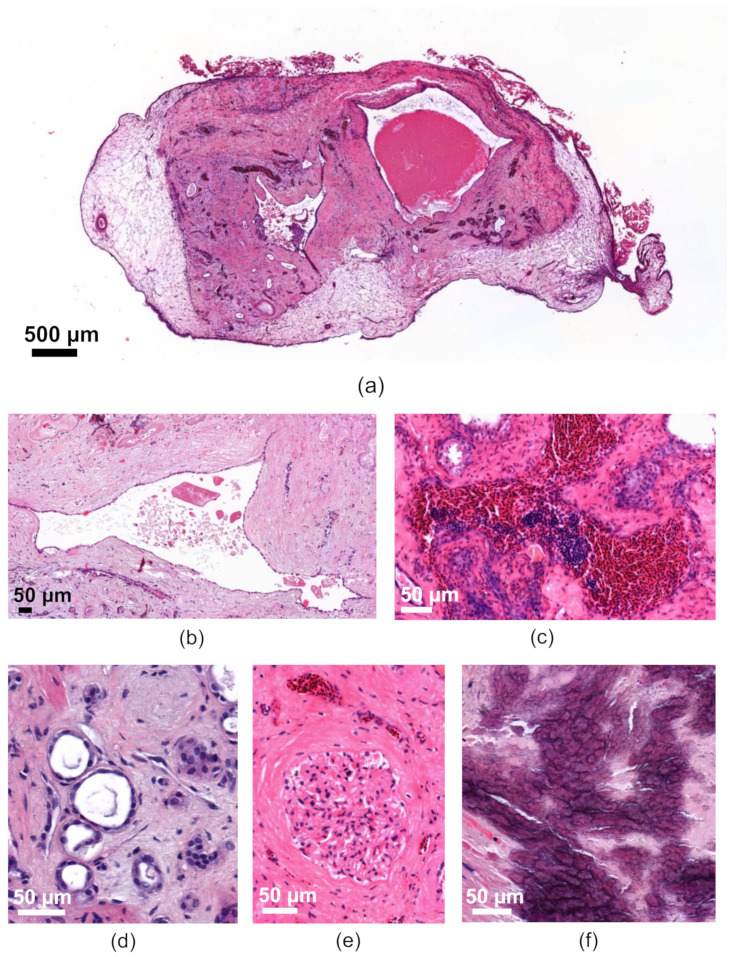
Representative H&E staining of human cystic tissue after removal from the CAM; (**a**) overview showing cystic tissue with two bigger cysts within the renal tissue surrounded by the CAM; (**b**) cyst with a typically flat epithelium and intraluminal debris; (**c**) accumulation of erythrocytes in a cyst; (**d**) vital parts of the tubule system; (**e**) vital glomerulus; and (**f**) bradytrophic calcifications.

The engrafted human renal cysts remained vital on the CAM for more than one week. H&E staining showed a smooth transition between the CAM and the engrafted tissue as a sign of successful attachment (Figure 11a). The cyst-lining cells of bigger cysts displayed a typical flat epithelium (Figure 11b). We also observed the characteristic heterogeneity of cysts with different sizes, with some cysts showing intraluminal debris or blood (Figure 11a–c). Moreover, characteristic staining patterns of the tubule system could be detected (Figure 11d) and glomeruli characterized by sclerosis, but without necrosis, appeared vital and often contained eosinophil granulocytes (Figure 11e). Many cysts also exhibited bradytrophic calcifications, a characteristic that is also found in freshly isolated human cystic kidney tissue, and which is usually regarded as a sign of progressive inflammation in cystic tissue (Figure 11f). Surprisingly, erythrocytes found in the cysts and tubules were often nucleated, which confirms that they originated from the chicken embryo (Figure 11c).

**Figure 12:**
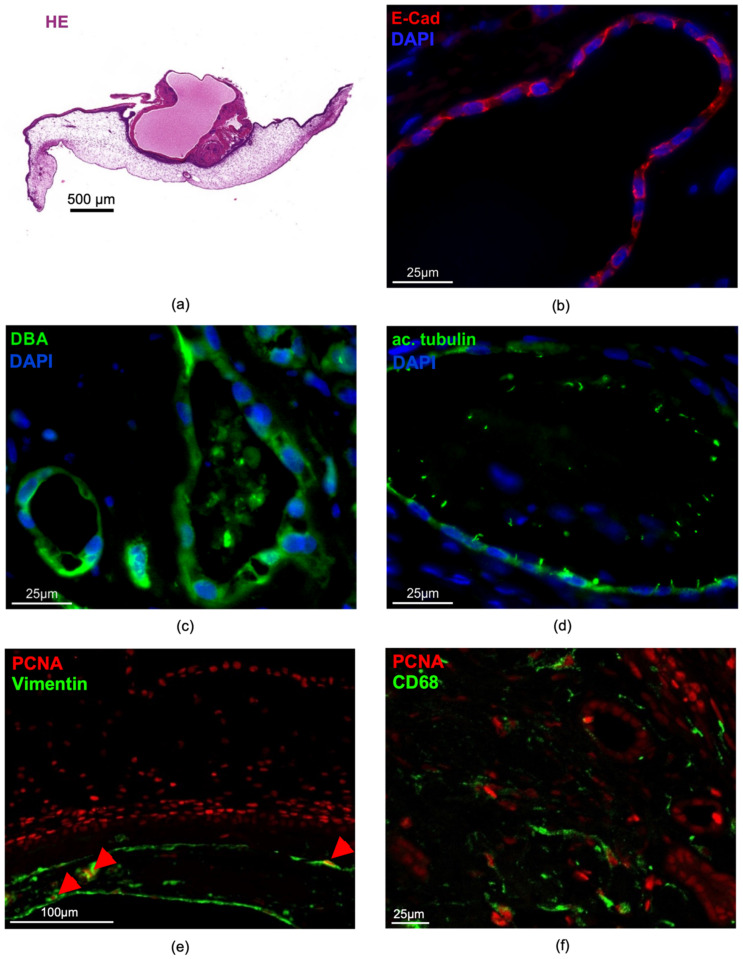

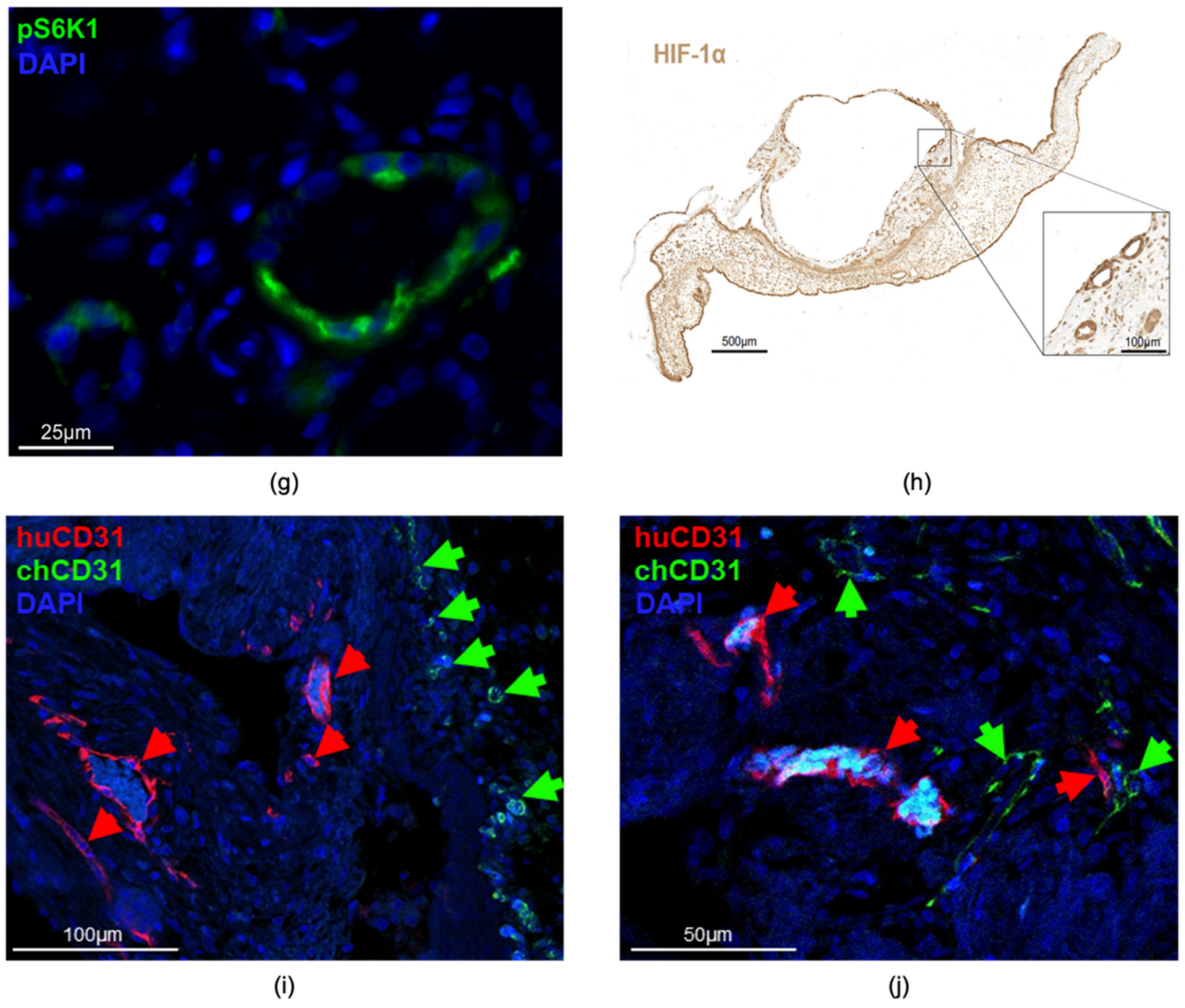
Representative histological and immunohistological staining of human cystic tissue after removal from the CAM; (**a**) overview of hematoxylin/eosin-stained cystic tissue, showing a big cyst within the renal tissue that is surrounded by the CAM; (**b**) vital parts of the cysts stained positive for E-cadherin; (**c**) Dolichos biflorus agglutinin (DBA) and (**d**) acetylated tubulin; (**e**) some of the vimentin-positive cells were also positive for the proliferating cell antigen (PCNA, red arrowheads); (**f**) CD68-positive macrophages and numerous PCNA-positive cells were observed within the cysts; (**g**) phosphorylated S6 kinase 1 (pS6k1) and (**h**) HIF-1α were detected in cyst-lining cells; (**i**) CD31-positive capillaries originating from human cysts (huCD31, red arrowheads) were clearly separated from chicken-derived CD31-positive capillaries (chCD31, green arrowheads) (**j**) or detected in close proximity. Nuclei were stained with DAPI (blue).

To characterize the human renal cystic tissue after growth on the CAM, we performed immunohistochemistry and immunofluorescence staining of the typical morphological features also in addition to signaling pathways that were seen to promote cyst enlargement (Figure 12). First, large parts of the human cystic tissue remained vital, except for only a few areas that appeared necrotic at the end of the experiments (Figure 12a). Cysts within the vital parts stained positive for E-cadherin, representing a typical epithelial cell marker (Figure 12b). In addition, many cysts were positive for Dolichos biflorus agglutinin (DBA), a marker which indicates that these cysts originated from distal tubules and collecting ducts, which are supposed to be the main source of cyst development in ADPKD (Figure 12c). Furthermore, primary cilia, a typical feature of tubule epithelial cells, were detectable in the cyst-lining cells one week after growth on the CAM (Figure 12d). Cyst growth has been shown to be mediated by the proliferation of the cyst-lining cells, which was confirmed on the CAM through a significant number of PCNA-positive cells (Figure 12e). In addition, mTOR signaling significantly contributes to cyst enlargement in ADPKD. We consistently found phosphorylated S6 kinase 1 (pS6k1), a well-characterized downstream effector of mTOR, in the cyst-lining cells one week after engraftment (Figure 12g). In addition, interstitially localized macrophages also contribute to cyst growth and were found in the human cystic tissue (Figure 12f). Cyst growth leads to the compression of intact tissue, leading to hypoxia, which was evident in our model as shown by HIF-1α staining in the cyst epithelium (Figure 12h). CD31 of human and chicken origin could be clearly distinguished between the cysts and the CAM; therefore, the staining of the respective CD31 was usually restricted to the tissue of origin (Figure 12i). However, areas where chicken CD31 and human CD31 were detected in the same area were sporadically found (Figure 12j).

## 4. Discussion

So far, it has not been possible to successfully cultivate adult mouse kidney tissue in an in vivo model due to the lack of oxygen supply and nutrition via diffusion into the vulnerable tissue. However, this would be of large value for the in vivo investigation of physiological as well as pathophysiological mechanisms (e.g., repair mechanisms after damage). Therefore, we have put forward a protocol that enables the cultivation of mature mouse kidney slices on the CAM to study kidney (patho-) physiology. We presumed that the cultivation of entire adult kidneys would be challenging because of the supply of nutrition on the CAM. Our experiments therefore specifically aimed to use mouse kidney slices. We further demonstrated that covering the renal tissue with fleece is a possible option for the successful engraftment and maintenance of tissue vitality. Although the cutting technique may not play a significant role in maintaining the vitality of the kidney slices, the thickness of the slices should be kept as thin as possible. Thus, our approach offers a plethora of kidney-related analyses that extend far beyond polycystic kidney disease.

According to the histological analyses, many parts of the mouse kidney appeared vital, including the glomeruli, proximal tubules, and collecting ducts. However, vulnerable parts of the nephron, such as the thick ascending limb and the distal tubules, were significantly damaged. Similar damage occurs after ischemia/reperfusion injury, for example [28]. This might be due to an insufficient supply of nutrition to these parts of the murine kidneys, particularly if they were not closely connected to the vasculature of the CAM or exposed to the outside. To improve the supply of nutrition to the vulnerable parts of the kidneys, different concentrations of the RPMI medium as well as other types or compositions of nutrient media could be applied to the tissue, and alternative materials to the autoclaved pieces of fleece could be compared. Even though the histology of the mouse kidneys showed vital tissue in most parts, ultrastructural analyses, including electron microscopy, will be needed for future studies to consolidate the histological findings and to evaluate the functionality of the mouse kidney more in-depth.

The successful cultivation of adult vital kidney tissue could ultimately provide a model for studying acute renal damage and also facilitate the examination of chronically damaged kidney tissue (e.g., fibrotic tissue) on the CAM using the proposed live-imaging and live-follow up analyses. The model also contributes to a more sophisticated differentiation between endogenous and exogenous mechanisms with regards to their contributions to inflammation and fibrosis after different forms of damage.

Interestingly, the human cystic kidney tissue showed less damage compared to the murine kidney slices, which was indicated by the ongoing cell proliferation and preserved capillarization. The presence of embryonic chicken erythrocytes in the human cysts, as shown in the H&E-stained sections and especially demonstrated in the lumen of the human CD31-positive vessels (Figure 11 and Figure 12), suggests that the human tissue was supplied by blood from the chicken embryo, which indicated a connection between these two blood vessel networks. In addition, the close spatial proximity of the chicken and human-derived CD31-positive capillaries also indicated an invasion of the chicken vessels into the human tissues, which supports the potential connection of the two vascular systems (Figure 12). Interestingly, we recently made the same observation of circulating chicken erythrocytes within primary human breast cancer tissue after growth in the CAM model [26].

At the moment, it is still unclear if the tissue vitality of human tubular structures and glomeruli depends on the invasion of chicken erythrocytes into the cystic tissue as observed by H&E staining. Further research is needed to assess this information. Interestingly, the human ADPKD tissue seemed less vulnerable than the mouse kidney slices when cultivated on the CAM, as seen in histological and immunofluorescence staining of the cystic tissues, which depicted less-damaged renal structures (Figure 11 and Figure 12) than in the staining of the murine kidney slices (Figure 5a). Whether this is due to differences in the nutrient supply of cystic versus healthy tissue or due to reduced requirements of ADPKD tissue remains elusive at the moment.

For the first time, we have shown the successful engraftment of primary renal tissue derived from eight different ADPKD patients onto the CAM. Apart from utilizing tissue from different patients, we also cultivated multiple cysts from the same renal tissue sample, but from different parts of a polycystic kidney. This allows us to show that the proliferation and cyst growth were not specific to the entire kidney but have a certain variability within a cystic kidney.

After cultivating the human renal cystic tissue on the CAM for one week, we observed: (1) the maintenance of the vitality of the tissue, (2) preservation of the typical morphology of the primary cystic tissue, and (3) growth of the preexisting cysts as well as (4) the formation of new cysts. Thus, we can use the CAM model to gain a better understanding of cyst growth as well as cyst formation in human tissue. However, the model may also be useful for the testing of already existing therapies. For example, tolvaptan^®^ does not provide the same results in every single patient. Given the heterogeneity of human samples, this may help to dissect the underlying mechanisms leading to different responses upon therapeutic interventions.

The next step for the further development of the proposed model will be testing the effects of different substances on cyst growth and the sensitivity of human polycystic kidney tissue in the CAM model. Here, standard medications of chronic kidney disease patients as well as new molecular targets with promising growth-suppressing effects in ADPKD mouse models could be used.

Apart from cAMP-activated chloride secretion through CFTR, a second mechanism facilitating cyst growth and formation was identified with Ca^2+^-stimulated chloride secretion by TMEM16A [13]. Recent data have consistently shown that the cyst growth in ADPKD mouse models was inhibited through different pharmacological inhibitors of the Ca^2+^-activated chloride channel [9]. Therefore, the inhibition of the TMEM16A channel through FDA-approved drugs such as niclosamide and benzbromarone, as well as the specific TMEM16A inhibitor Ani9, should be investigated regarding their effects on the growth of renal cystic tissue in the CAM model. In addition, the relevance of CFTR-mediated chloride secretion could be evaluated by testing the effects of CFTR inhibitors established in mouse models [11] on the growth of different human cystic tissues. Even though we acknowledge the fact that the assessment of possible drug concentrations may be impaired because of the lack of in vivo permeability, the protocol could help to gain first insights into the effects of drugs on human renal cyst growth. So far, a variety of substances have been evaluated in different types of patient-derived tissues and cell-line tumors in the CAM model, including gliomas [29], glioblastomas [21], lung cancer, and prostate cancer [30]. In addition, the CAM model could be used to compare local application and intravenous injection into the blood vessels of the CAM [21]. Despite the lack of glomerular filtration in the CAM model, we believe that the blood supply through the CAM vessels would enable insight into the possible effects of drugs. Since the plasma level and protein binding of multiple substances are also well-known, this could subsequently lead to approximations of effective concentrations without long-running experiments, as they might be performed in other in vivo models. For instance, one could measure the blood flow in the CAM by means of laser speckle contrast imaging (LSCI) [25] and add it to the known pharmacodynamics of the applied substance in addition to the volume of the cystic tissue. Another possibility would be the measurement of the weight or the volume of the cystic tissue to gain dose response curves, which has been utilized in a similar study for the assessment of drug concentrations for tumors on the CAM [31]. Furthermore, the successful grafting of kidney organoids that were derived from human pluripotent stem cells on the CAM illustrates the possibility of establishing functional blood circulation to the tissue [32,33]. However, it should also be evaluated how secondary effects of the treatment, such as a reduced blood supply to the human tissue due to damages of the CAM, contribute to the overall effectiveness of the substances. Studies on potential therapeutic agents in the CAM model should therefore include a sufficient control group that allows an evaluation of cytotoxic effects on the chick embryo.

Despite numerous advantages of the CAM model, including the cost-effectiveness and the lack of innervation of the CAM in the early days of development, this method must still be seen critically and ethical principles must be strictly observed. Furthermore, compared to conventional animal models, the CAM model is characterized by its limited examination time. However, it is generally possible to transplant the cysts onto a new generation of eggs after they have been removed from the CAM, thus allowing them to be examined over a time span of several weeks [24]. The extent to which the transplanted cysts remain vital for analytical purposes has not been clarified yet.

The volume and weight of engrafted cysts were monitored using different methods. Three-dimensional volume measurements utilizing a digital VHX-7000 microscope (Keyence) have proven to be practicable and highly reproducible regarding the ex ovo volume measurement of cystic tissue. Furthermore, a correlation between the volume and weight of ADPKD tissue samples could be shown with this technology. Although three-dimensional volume measurements could also be used for the analysis of in ovo volumetric data during the cultivation period, they are not suitable for the measurement of tissue that grows underneath the CAM. Therefore, we performed supplementary CT-based volume measurements, which allowed for the in ovo detection of cystic parts even underneath the CAM. Furthermore, the CT scan enabled the analysis and quantification of the calcifications. To test the accuracy of CT-based measurements, we compared the data acquired with this technique to the results of the microscopic volumetry method. We observed that the CT-based measured volume was generally higher than the ex ovo volumetric data, which could be explained by the difficult distinction between low-contrast cystic tissue and parts of the CAM in the CT scans. Nevertheless, the CT-based volumetry technique presents a suitable method for the in ovo examination of the volume of ADPKD tissue samples grafted onto the CAM, as well as for additional verification of the cyst volumes.

Cyst calcification is a common phenomenon of ADPKD, predominantly in the advanced stages, and may be due to previous cyst hemorrhage [34,35]. So far, it is still not clear whether the calcifications, which appear hyperdense in the CT scans, are already present to a microscopical extent in the ADPKD kidney before the engraftment of cystic material on the CAM and to what extent the calcifications increase during the growth period on the membrane. No calcifications were detectable in histologically stained sections of the mouse tissue cultured on the CAM. Since chicken erythrocytes invaded the human tissue, simply the presence of blood can be excluded as a promoter of calcification. However, inflammation, which is often present in ADPKD tissue, may be a prerequisite for calcification. Calcification may be a surrogate marker for inflammation, and therefore may be of prognostic value. However, further studies are needed to evaluate this. In this regard, the proposed protocol might be of value for the assessment of calcified cystic tissue.

## 5. Conclusions

For the first time, this study showed that human renal cystic tissue can be successfully grafted onto the CAM. We established a protocol for the monitoring of the growth processes of renal cystic tissue derived from ADPKD patients through measurements of volume, weight, and angiogenesis. For this reason, the CAM model might be a sufficient addition to other in vivo models and might allow for a better translation of data from in vitro experiments into the in vivo setting. In addition, the histological analyses of mouse renal tissue showed evidence of tissue vitality after one week of growth on the CAM. In summary, the human-tissue-based cyst model might not only be of additional value in assessing the pathophysiology of cyst growth and testing promising treatment strategies, but might also contribute to the reduction of animal experiments according to the 3R-principles (“reduce, replace, refine”).

## Data Availability

Data is contained within the article. The data presented in this study are available in A 3D In Vivo Model for Studying Human Renal Cystic Tissue and Mouse Kidney Slices.
